# Prevalence of *Chlamydia trachomatis* Infection, Serovar Distribution and Co-Infections with Seven High-Risk HPV Types among Italian Women with a Recent History of Abnormal Cervical Cytology

**DOI:** 10.3390/ijerph16183354

**Published:** 2019-09-11

**Authors:** Marianna Martinelli, Rosario Musumeci, Alberto Rizzo, Narcisa Muresu, Andrea Piana, Giovanni Sotgiu, Fabio Landoni, Clementina Cocuzza

**Affiliations:** 1 Department of Medicine and Surgery, University of Milano-Bicocca, 20900 Monza, Italy; marianna.martinelli@unimib.it (M.M.); rosario.musumeci@unimib.it (R.M.); a.rizzo@campus.unimib.it (A.R.); fabio.landoni@unimib.it (F.L.); 2 Department of Medical, Surgical and Experimental Sciences, University of Sassari, 07100 Sassari, Italy; narcisamuresu@outlook.com (N.M.); piana@uniss.it (A.P.); gsotgiu@uniss.it (G.S.); 3 ASST Monza, San Gerardo Hospital, 20900 Monza, Italy

**Keywords:** *Chlamydia trachomatis*, *Chlamydia trachomatis* serovars, human papillomavirus, *Chlamydia trachomatis*, HPV co-infection

## Abstract

*Chlamydia trachomatis* (Ct) and human papillomavirus (HPV) are the most prevalent sexually transmitted infections throughout the world. Despite the serious complications associated with chronic Ct infections in sexually active women, a screening program is not yet available in Italy. Moreover, HPV/Ct co-infections are also known to occur frequently, increasing the risk of HPV-induced carcinogenesis. The aim of this study was to evaluate the prevalence of Ct infections, the distribution of Ct serovars, and the incidences of Ct/HPV co-infections among women with a recent history of abnormal cervical cytology. Cervical samples were collected from 199 women referred for a gynecological visit following an abnormal Pap test results. All samples were tested for the presence of Ct and HPV DNA using real-time PCR assays; Ct typing of positive samples was performed by PCR–RFLP (restriction fragment length polymorphism) targeting the *ompA* gene. A high percentage of these women (12.8% and 21.7% with or without abnormal cytology on “retesting”, respectively) were found to be Ct positive. Serovar F was the most prevalent type in Ct positive women, followed by E and K. Ct/HPV co-infections were detected in 7% (14/199) of enrolled women, with HPV-16, HPV-51, and HPV-52 being most frequently identified in co-infections. This study provides new epidemiological data on the prevalence of Ct and associated HPV infection in women with a recent history of abnormal cervical cytology in Italy, where notification of cases is not mandatory.

## 1. Introduction

*Chlamydia trachomatis* (Ct) is the most prevalent sexually transmitted bacterial agent worldwide. The World Health Organization (WHO) estimated that in 2013, 131 million new cases of chlamydia infections occurred among adults and adolescents aged between 15 and 49 years worldwide, with a global incidence rate of 38 per 1000 female and 33 per 1000 males [1]. In 2015, 394,163 cases of chlamydia infection were reported in 27 European Union/European Economic Area (EU/EEA) Member States, with the largest proportion of cases among 20–24-year-olds, which accounted for 39% of all cases [2].

*Chlamydia trachomatis* can be differentiated into several serological variants (serovars) with a number of variants being identified on the basis of the serological reactivity of the epitopes of the major outer membrane protein (MOMP) [3]. These serovars belong to two major biological variants (biovars): lymphogranuloma venereum (LGV) and the noninvasive trachoma epitheliotropic biovar. The LGV biovar (L1, L2, L3) causes invasive sexually transmitted diseases (STDs) [4]. The trachoma biovar (A, B, Ba, C, D, Da, E, F, G, H, I, Ia, J, Ja, K) is responsible for the ocular disease, termed trachoma, and urogenital pathologies, including pelvic inflammatory disease (PID) and infertility [5].

Despite the serious sequelae associated with this infection, there are no exhaustive epidemiological data about the incidence and distribution of different serovars of *Chlamydia trachomatis* in Italy. Some studies have found that *Chlamydia trachomatis* prevalence rates range from 1.8 to 10.4% among sexually active women, depending on the study population, the geographical area, and the methods used for detecting infection [6,7,8,9,10]. Although the infection is treatable, its asymptomatic nature (more than 80% of women with infection) makes it difficult to recognize and treat an unscreened population. Furthermore, the high prevalence of asymptomatic infection in young people represents an important reservoir for transmission.

Moreover, several studies have reported an increased risk of human papillomavirus (HPV) infection among *Chlamydia trachomatis* positive women. Chlamydial infection may lead to epithelial disruption and facilitate the entry of HPV, or it may impair the immune response favoring the persistence of HPV [11].

The aim of the present study was to evaluate the prevalence of *Chlamydia trachomatis* infections, serovars distribution, and Ct/HPV co-infection in women with a recent history of an abnormal Pap test result, in order to better estimate the burden of these infections in women with higher risk of developing cervical dysplasia.

## 2. Material and Methods

### 2.1. Study Design and Sample Collection

One hundred ninety-nine women were consecutively enrolled in the study during an outpatient referral visit at the Gynaecology Clinic of San Gerardo Hospital, Monza (Lombardy region, Italy) between 2009 and 2011 following an abnormal Pap smear found as part of their routine participation in local cervical cancer screening programs. Patients’ referral visits and enrollment generally took place 2 to 6 months following the initial abnormal Pap smear result.

The study protocol (Protocol: 08/UNIMIB-HPA/HPV1; n. 1191) was approved by the Ethics Committee of San Gerardo Hospital, Monza, Italy. All recruited subjects provided written informed consent to participate in the study. All women underwent a routine gynecological examination and a repeat Pap smear at the time of enrollment; an additional cervical sample was also collected during the visit for high-risk HPV (hr-HPV) and *Chlamydia trachomatis* DNA detection. Repeat cervical cytology was routinely performed by the hospital, and findings were assessed according to the 2001 Bethesda System for cervical cytological reporting [12].

Cervical samples were tested for hr-HPV and *Chlamydia trachomatis* at the Clinical Microbiology Laboratory of the Department of Medicine and Surgery, University of Milano-Bicocca, Italy.

Cervical cytology samples were collected using the Abbott cervicollect specimen collection kit (Abbott) and transported in ThinPrep^®^ PreservCyt^®^ Solution (HOLOGIC™). Fifteen milliliters of cervical sample was centrifuged at 2000 rpm for 15 min at 4 °C to spin down cervical cells. Cell pellets were suspended in 2.5 mL of PBS, and 5 aliquots of 500 µL for each sample were stored at −80 °C prior to testing. One aliquot was subsequently used for DNA extraction.

### 2.2. DNA Extraction and High-Risk HPV Detection

DNA extraction from a 500 µL cell pellet aliquot of cervical sample was carried out using CLART^®^ HPV2 Extraction–Purification and Amplification kit (Genomica) according to the manufacturer protocol.

HPV DNA amplification was carried out using previously described “in-house” real-time TaqMan PCR assays [13,14,15,16]. The hr-HPV genotype-specific assays allow detection and quantification of HPV 16, 18, 31, 33, 45, 51, and 52 DNA. Amplification was performed using ABI PRISM device (7900 SDS; Applied Biosystems, Foster City, CA, USA). The thermal cycling conditions, optimized to obtain the best amplification kinetics under the same temperatures and composition of reaction mixture, consisted of the following thermal profile: 2 min at 50 °C, 10 min at 95 °C, and 40 cycles of 15 s at 95 °C and 1 min at 60 °C. All HPV real-time quantitative TaqMan PCR assays were previously validated by participation to WHO HPV Laboratory Proficiency Lab-Net 2014 and 2017.

### 2.3. Chlamydia trachomatis Detection and Typing

*Chlamydia trachomatis* DNA amplification was carried out using previously described real-time PCR Evagreen^®^ assays [17]. Amplification was performed using ABI PRISM device (7900 SDS; Applied Biosystems). The thermal cycling conditions consisted of the following thermal profile: 2 min at 95 °C and 40 cycles of 5 s at 95 °C and 30 s at 60 °C.

Typing of clinical specimens required the amplification of *ompA* gene by PCR using specific primers. The fragment was typed using restriction fragment length polymorphism (RFLP) analysis as previously described [18].

### 2.4. Statistical Analysis

Qualitative and quantitative variables were summarized with absolute (relative) frequencies and medians (interquartile ranges, IQR), respectively. A chi-squared or Fisher exact test was performed for testing qualitative variables. A two-tailed *p*-value less than 0.05 was considered statistically significant. The statistical software STATA version 15 (StatsCorp, TX, USA) was used for all statistical computations.

### 2.5. Ethics Approval and Consent to Participate

All subjects provided written informed consent to participate to the study. The study protocol was approved by the Ethics Committee of San Gerardo Hospital, Monza, Italy (Protocol: 08/UNIMIB-HPA/HPV1; n. 1191).

## 3. Results

### 3.1. Cervical Cytology Analysis

**Figure 1:**
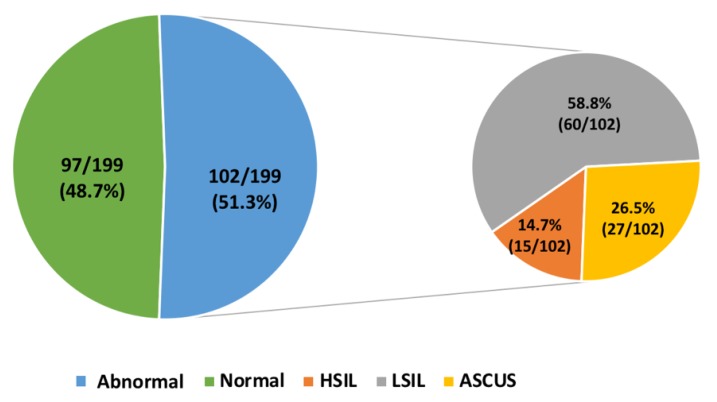
Cervical cytology results on “retesting” at enrollment.

One hundred ninety-nine consecutive women with a recent history of abnormal cervical cytology were recruited. An abnormal Pap test at enrollment (“retesting” result) was detected in 51.3% (102/199) of women (median age 31 years, IQR: 26–42 years): 14.7% (15/102) showed a high-grade squamous intraepithelial lesion (HSIL), 58.8% (60/102) a low-grade squamous intraepithelial lesion (LSIL), and 26.5% (27/102) atypical squamous cells of undetermined significance (ASCUS). Normal cervical cytology was detected on “retesting” in 48.7% (97/199) of recruited women (median age 35 years, IQR: 27–44 years) with a previously documented abnormal Pap test in the preceding 2–6 months (Figure 1).

### 3.2. Chlamydia trachomatis Detection

Overall, 12.8% (13/102) of retested women with abnormal cytology were found to be *Chlamydia trachomatis* positive. At enrollment, 11.1% (3/27) had ASCUS, 11.7% (7/60) LSIL, and 20% (3/15) HSIL (*p*-value: 0.64). Women aged less than 32 years showed a prevalence of *Chlamydia trachomatis* infection of 13.0% (7/54), whereas those aged ≥32 years showed a prevalence of infection of 12.0% (6/48) (*p*-value: 0.94). Serovar was determined in 12 out of 13 *Chlamydia trachomatis* positive samples. The most prevalent serovar detected was serovar F in 33.3% (4/12) of positive samples, followed by serovar K (3, 25.0%), E (2, 16.7%), and D (2, 16.7%).

**Figure 2:**
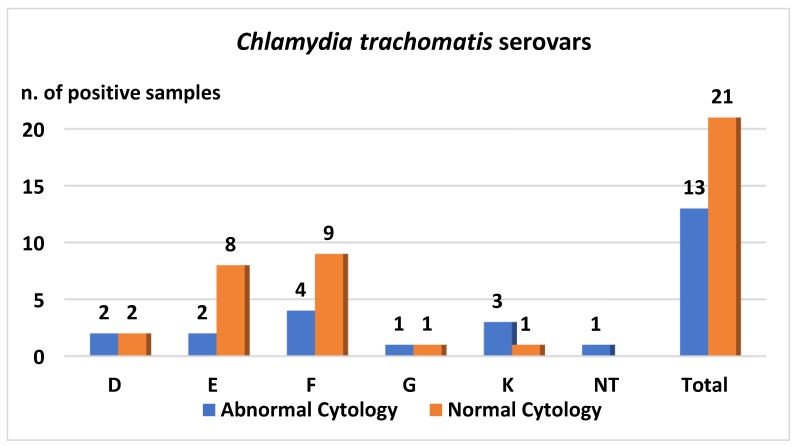
*Chlamydia trachomatis* (Ct) serovar distribution based on cervical cytology result.

In women with normal cytology on “retesting”, *Chlamydia trachomatis* was detected in 21 women (21/97, 21.7%); 13 of these were aged <32 years. Comparison between women with an age less than 32 years (13/39, 33.3%) and those aged ≥32 years (8/58, 13.8%) infected with *Chlamydia trachomatis* was statistically significant (*p*-value: 0.03). The predominant serovar identified in this population was again serovar F (9/21, 42.9%), followed by serovar E (8/21, 38.1%). Data regarding the different serovars detected are reported in Figure 2.

Graph shows the Ct serovar identified in cervical samples of hr-HPV positive women.

### 3.3. HPV Detection

**Figure 3:**
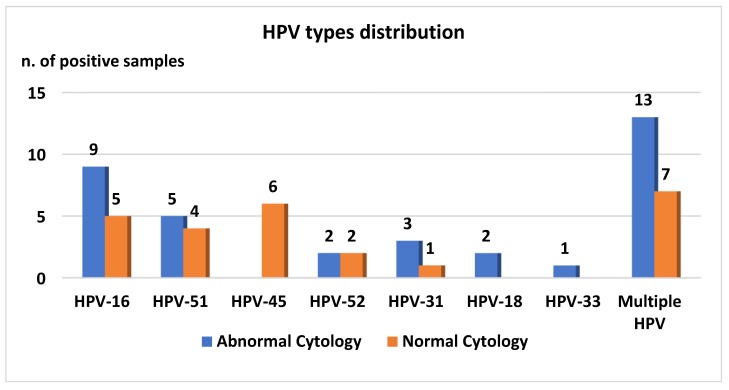
Human papillomavirus (HPV) genotypes distribution based on cytological result.

Overall, 34.3% (35/102) of retested women with abnormal cervical cytology were found to be positive for the detection of one or more of the seven hr-HPV types investigated, and 34.3% (12/35) of HPV DNA positive women showed infection with multiple HPV types. The most prevalent types were HPV-16 (16, 15.7%), HPV-51 (14, 13.7%) and HPV-52 (8, 7.8%) (Figure 3). HPV DNA was detected in 53.3% (8/15) of women with HSIL, 35.0% (21/60) with LSIL, and 22.2% (6/27) with ASCUS (*p*-value: 0.12).

HPV infection was observed in 25 (25/97, 25.8%) women with normal cervical cytology, and seven (7/97, 7.2%) were shown to have infection with multiple HPV types. HPV-45 (9, 9.3%) and HPV-16 (8, 8.3%) were the most prevalent types identified (Figure 3).

Graph shows the HPV types identified in cervical samples of HPV positive women.

### 3.4. Chlamydia Trachomatis and HPV Co-Infection

*Chlamydia trachomatis* and HPV co-infections, with one or more of the investigated seven high-risk HPV types, were detected in 7% (14/199) of patients enrolled.

In particular, Ct/HPV positivity was found in 4.9% (5/102) of women with cervical lesions; 3/54 (5.6%) women were less than 32 years old and 2/48 (4.2%) women were aged ≥32 years. Three out of seven women (42.9%) showed LSIL on cervical cytology “retesting” at enrollment; the other two were found to have ASCUS and HSIL.

Nine (9/97, 9.3%) women with normal cervical cytology on “retesting” were found to be Ct/HPV co-infected, and five (5/39; 12.8%) of them were younger than 32 years.

## 4. Discussion

This study reports the results obtained from an analysis carried out on a population of women with recent history of abnormal cervical cytology whose ages ranged from 16 to 73 years old (median age 31 years, IQR: 26–42 years). The percentage of *Chlamydia trachomatis* infection demonstrated in the studied population (12.8% and 21.7% for women with or without abnormal cytology on “retesting”, respectively) was higher compared to that reported in previous studies conducted in Italy [7,19,20,21]. Foschi et al. reported an overall Ct prevalence of 8.1% in a male and female population referred to STI outpatient clinics, general practitioners, or gynecology clinics [7]. A Ct positivity of 4.5% among asymptomatic women with no cytological lesions was indicated by Seraceni and colleagues, whilst Mancini et al. detected a Ct positivity rate of 10%, with 9% of Ct and HPV co-infections in a group of women with cervical dysplasia [19,20]. 

A higher percentage of *Chlamydia trachomatis* positivity was found among women aged less than 32 years (*p*-value: 0.03). This result is in agreement with the majority of previous surveys, which reported sexually transmitted infections to be more common in younger women [2]. 

Unlike data reported in other previous Italian studies (2011–2014, 2007–2009), which described serovar E as the most common Ct serovar detected [7,22], in this study, the most prevalent serovar identified was F, followed by E and K. Moreover, in another study performed in Italy between 2002 and 2003, in which women with an abnormal Pap smear were enrolled, the two most prevalent serovars described as circulating at that time were G (25%) and H (25%), followed by F (19%), D (12%), E (12%), and K (7%) [20]. Although the difference in the prevalence of F and E serovars observed in the present, more recent study, is not statistically significant, the results suggest a possible change in distribution of serovars over time. This change over time was also observed by Wikström et al. in Finnish women [23].

We also did not observe an association between abnormal Pap smear and the presence of Ct. In fact, results showed a higher prevalence of *Chlamydia trachomatis* in women with normal cytology. Future longitudinal studies with a larger number of enrolled women would be useful to better understand this potential association. 

More than 34% of women with abnormal cytology on “retesting”, as well as more than 25% of those with a negative Pap-smear, were found to be positive for the detection of one or more of the 7 hr-HPV types investigated. These results suggest that the virus could continue to be present at the cervical site even when the cytological abnormality has regressed. The relatively low prevalence of HPV positivity observed in this study in women with two consecutive abnormal Pap smears can be explained by the detection of only seven hr-HPV types, in accordance with the results of a previously-published study using the same HPV test [24]. *Chlamydia trachomatis* and HPV co-infections in that study were found to occur in 4.9% and 9.3% of patients with and without cervical cytology abnormalities, respectively. The prevalence of both HPV infections and HPV/Ct co-infections found in our study is included in the spectrum of rates previously reported in the literature [9,10,14,19,20,21,24,25,26,27,28,29,30]. This variability depends on the type of population studied, the methods used for HPV detection, and the HPV types being investigated. In this study, seven of the most common hr-HPV types were studied, and the most prevalent genotype detected was found to be HPV-16, followed by HPV-51. As expected, HPV-16 was the most common type identified in the cervical sample of women with cervical dysplasia. On the other hand, the high prevalence of HPV-51 is infrequent since this genotype accounts for 1% of invasive cervical cancers based on the estimates from international prevalence studies. Nevertheless, this result was also reported in two other studies conducted in Italy, one describing HPV-51 as the second most prevalent type after HPV 16, detected in tissue samples of women with cervical cancer, and the other indicating HPV 51 as the third most frequent high-risk HPV type identified in young women (13–26 years old) [29,30].

It is important to consider some of the limitations of this study due to the small sample size, especially considering the number of women with HSIL, and the use of an HPV detection method that allowed the detection of only seven out of fourteen high-risk HPV types. However, this study provides updated information on the epidemiology of *Chlamydia trachomatis* infections in women followed up after an abnormal Pap smear. This is particularly important as notification of Ct cases is not mandatory, and screening programs for *C. trachomatis* infections are not presently available in Italy. Further studies are needed to better understand the role of HPV/Ct co-infection in the progression of cervical dysplasia as well as the circulation and pathogenic role of specific Ct serovars in order to improve well-organized control and prevention strategies.

## 5. Conclusions

This study provides new data regarding *Chlamydia trachomatis* prevalence and different serovar circulation among Italian women living in the Lombardy region with recent abnormal cytology results. A relatively high percentage of women with Ct infection alone or in combination with seven hr-HPV types was documented in the studied population. These findings warrant further investigation through longitudinal studies to determine the clinical relevance of these often asymptomatic infections, in terms of the potential associated complications of chronic infection as well as their role in the persistence of oncogenic HPVs.

## Abbreviations

Ct	Chlamydia trachomatis
WHO	World Health Organization
EU/EEA	European Union/European Economic Area
MOMP	major outer membrane protein
LGV	lymphogranuloma venereum
STDs	sexually transmitted diseases
PID	pelvic inflammatory disease
HPV	human papillomavirus
RFLP	restriction fragment length polymorphism
PCR	polymerase chain reaction
